# Investigation of Cannabinoid Acid/Cyclodextrin Inclusion Complex for Improving Physicochemical and Biological Performance

**DOI:** 10.3390/pharmaceutics15112533

**Published:** 2023-10-26

**Authors:** Chulhun Park, Jieyu Zuo, Myung-Chul Gil, Raimar Löbenberg, Beom-Jin Lee

**Affiliations:** 1 College of Pharmacy, Jeju National University, Jeju 63243, Republic of Korea; chpark@jejunu.ac.kr; 2 Faculty of Pharmacy & Pharmaceutical Sciences, University of Alberta, Edmonton, AB T6G 2E1, Canada; zjieyu@ualberta.ca (J.Z.); raimar@ualberta.ca (R.L.); 3 College of Pharmacy, Ajou University, Suwon 16499, Republic of Korea; kilmch88@naver.com; 4 PLUTO Inc., Seongnam 13453, Republic of Korea

**Keywords:** cannabinoid acid, inclusion complex, spray-freeze-drying, methylated β-cyclodextrin, solubility, stability, permeability, anti-cancer activity

## Abstract

This study aimed to investigate the enhancement of cannabinoid acid solubility and stability through the formation of a cannabinoid acid/cyclodextrin (CD) inclusion complex. Two cannabinoid acids, tetrahydro-cannabinolic acid (THCA) and cannabidiolic acid (CBDA), were selected as a model drug along with five types of CD: α-cyclodextrin (α-CD), β-cyclodextrin (β-CD), γ-cyclodextrin (γ-CD), hydroxypropyl-β-cyclodextrin (HP-β-CD), and methylated-β-cyclodextrin (M-β-CD). Phase solubility studies were conducted using various types of CD to determine the complex stoichiometry. The preparation methods of the CD inclusion complex were optimized by adjusting the loading pH solution and the drying processes (spray-drying, freeze-drying, spray-freeze-drying). The drying process of the cannabinoid acid/M-β-CD inclusion complex was further optimized through the spray-freeze-drying method. These CD complexes were characterized using solubility determination, differential scanning calorimetry (DSC), field-emission scanning electron microscopy (FE-SEM), X-ray diffraction (XRD), and ^1^H NMR spectroscopy. DSC, XRD, and FE-SEM studies confirmed the non-crystalline state of the cannabinoid acid/CD inclusion complex. The permeation of THCA or CBDA from the M-β-CD spray-freeze-dried inclusion complex was highly improved compared to those of cannabis ethanolic extracts under simulated physiological conditions. The stability of the cannabinoid acid/M-β-CD inclusion complex was maintained for 7 days in a simulated physiological condition. Furthermore, the minimum inhibitory concentration of cannabinoid acid/M-β-CD inclusion complex had superior anti-cancer activity in MCF-7 breast cancer cell lines compared to cannabinoid acid alone. The improved physicochemical and biological performances indicated that these CD inclusion complexes could provide a promising option for loading lipophilic cannabinoids in cannabis-derived drug products.

## 1. Introduction

The pathophysiology and therapeutic potential of cannabinoids in various diseases have gained significant interest in recent years [1,2,3,4]. Most cannabis-derived drug products are commercially in oral dosage forms and only use tetrahydrocannabinol (THC) and cannabidiol (CBD) as model drugs [5]. The bioavailability of THC and CBD is limited owing to their lipophilicity, instability, and extensive metabolism in the liver, with only 13–19% being effectively utilized [6]. Cannabis-derived products containing only THC or CBD as an active pharmaceutical ingredient often incorporate surfactants or ethanol in the composition and manufacturing processes, leading to chemical instabilities, irritating mucosal issues, and discouraging long-term intake [7,8,9]. Specifically, the suspended oil, ethanol, and lipid-based surfactants induced high inter-subject variability in cannabinoid pharmacokinetics [9,10]. Therefore, the bioavailability of cannabinoids should be considered by decreasing the inter-subject variation in various patient groups, taking into account the effects of excipients and routes of administration [9,11].

Despite these challenges, studies have demonstrated that tetrahydro-cannabinolic acid (THCA) and cannabidiolic acid (CBDA) possess pharmacological activities, including anti-inflammatory, anti-epileptic, and anti-cancer effects [12,13,14,15,16]. Additionally, THCA and CBDA have been associated with the biological functions of inhibiting cyclooxygenase (COX) and cytokine (TNF-α, Interleukin), indicating their potential utility in cancer treatment [17,18]. THCA and CBDA are the primary components of certain cannabis plants, but they have been overshadowed by their derivatives THC and CBD. During cannabis plant growth, THCA and CBDA exist primarily in their acidic forms. Subsequently, cannabinoid acids in the extraction form can be readily decarboxylated when exposed to the extraction and pre-treatment system, rendering them effective as therapeutic agents [19]. Specifically, cannabinoid acids, such as CBDA and THCA, have been highlighted as promising candidates. However, the chemical instability and low water solubility of cannabinoid acids pose significant challenges to their effectiveness as anti-cancer agents. Therefore, further investigation of cannabinoid acid should be performed to verify its anti-cancer efficacy and be accompanied by the formulation of development strategies.

Cyclodextrins (CDs) are oligosaccharides obtained from the biodegradation of starch using glucano-transferase enzymes in three different forms: α-cyclodextrin (α-CD), β-cyclodextrin (β-CD), γ-cyclodextrin (γ-CD). CDs are widely used as complexing agents, and their specific structure enables the encapsulation of water-insoluble molecules in their internal cavities via inclusion complexation to improve the physicochemical properties and bioavailability of poorly water-soluble drugs [20]. CD-based inclusion complex formulations can load various drug molecules via non-covalent host–guest molecular interactions [21]. CD complexation also offers many pharmaceutical advantages for localizing drug molecules in the narrow absorption sites and reducing drug-induced irritation [22].

CD complexation is a versatile pharmaceutical strategy for modulating the permeation pathway while maintaining the biocompatibility of hydrophobic drug molecules [23]. Several formulation trials of CD molecules have focused on delivering the neutral cannabinoid form [24,25,26,27,28]. Paudel et al. prepared a ternary mixture of PEG400 and ethanol system [29]. They used only 1% dimethyl-β-CD as a permeation enhancer to improve the nasal absorption of CBD [29]. Additionally, three types of CDs were investigated for the structural activity of CBD on the host–guest inclusion complex [30]. The combination of nabilone (a synthetic form of THC approved by the USFDA) with β-CD notably enhances water solubility and absorption in the body [31]. The THC complex with methylated β-cyclodextrin (M-β-CD) was also structurally elucidated [28]. While previous studies have predominantly focused on isolated or synthesized cannabinoids [32,33,34], our research uniquely emphasized naturally derived cannabinoid acids. In fact, there were no detailed formulation and biopharmaceutical approaches to cannabinoid inclusion complexes utilizing CDs and their derivatives for various drug delivery systems.

This study aimed to investigate the inclusion complexation process for cannabinoid acids among various types of CD and to evaluate their physiochemical properties and cytotoxicity in MCF-7 breast cancer cell lines. Phase solubility studies of cannabinoid acid were performed to elucidate the solubilization capacity and complexation stability constant of each CD molecule. Inclusion complexes of cannabinoid acids with different types of CD were solidified using different pharmaceutical drying processes (spray-drying, freeze-drying, and spray-freeze-drying). The solid-state interactions of the cannabinoid acid/M-β-CD complex were characterized using differential scanning calorimetry (DSC) and X-ray diffraction (XRD) analysis. Morphological examination of each CD inclusion complex was performed using field-emission scanning electron microscopy (FE-SEM). No study examined the in vitro permeation of cannabinoid acids from differently prepared CD inclusion complexes. The effects of the manufacturing process on the in vitro permeation profiles of THCA and CBDA in the CD inclusion complex systems were evaluated. The stability profiles of cannabinoid acid in simulated physiological conditions (pH 7.4 PBS buffer and 37 °C) at 10% were also monitored for 7 days. Finally, in vitro cytotoxicity studies of the cannabinoid acid/M-β-CD inclusion complex were performed in comparison with the ethanolic extract of each cannabinoid acid to investigate the inhibitory anti-cancer activity in MCF-7 breast cancer cell lines.

## 2. Materials and Methods

### 2.1. Materials

The α-cyclodextrin (α-CD, Cavamax^®^ W6), β-cyclodextrin (β-CD, Cavamax^®^ W7), γ -cyclodextrin (γ-CD, Cavamax^®^ W8), and methylated-β-cyclodextrin (M-β-CD, Cavasol^®^ W7M pharma) were provided as a sample from Wacker Chemie GmbH (Burghausen, Germany). Hydroxypropyl-β-cyclodextrin (HP-β-CD), succinic acid, citric acid, sodium acetate, acetic acid, sodium carbonate, sodium bicarbonate, anhydrous ethanol, and methanol were purchased from MilliporeSigma (Oakville, ON, Canada). Two different cannabis ethanol extracts, THC-rich and CBD-rich cannabis extracts, were kindly provided by DDIC (Edmonton, AB, Canada) with support from XPhyto Therapeutics Inc (Vancouver, BC, Canada). The standard analytical solutions of cannabinoids were purchased from Cerilliant^®^ Corporation (Round Rock, TX, USA). The Alamar Blue and DMSO were purchased from Sigma Aldrich (St. Louis, MO, USA). All other analytical reagents and solvents used in this study were purchased from VWR International (Edmonton, AB, Canada).

### 2.2. HPLC Analysis of Cannabinoids

The cannabinoid concentrations were quantitatively analyzed using the reversed-phase (RP) column-based high-performance liquid chromatographic (HPLC) system [35]. The linearity, limit of detection (LOD), and limit of quantitation (LOQ) of THCA and CBDA were validated. Additionally, the precision and accuracy were determined for the quantitative analysis of THCA and CBDA. A Shimadzu CBM-20A HPLC system comprising an LC-10AD Pump and SIL-10A autosampler with an Ascentis^®^ C18 column (4.6 mm × 150 mm, particle size: 5.0 µm) and SPD-10AV dual-wavelength detector was used in this study. The isocratic method was applied to this analytical method for the HPLC analysis of cannabinoids. The mobile phase ratio was 72:28 acetonitrile:0.2% phosphoric acid in water. The chromatographic column was maintained at 30 °C. The flow rate of the mobile phase was kept at 1.0 mL/min. The detection wavelengths were set at 224 nm and 210 nm. The injection volume of each sample was 10 μL. Before running the HPLC system, the prepared mobile phase was sonicated and filtered with a 0.22 µm filter PTFE membrane (Millipore Sigma, Burlington, ON, Canada). The Lab Solutions system software was used for data processing and acquisition (version 3.1.05.9) [35].

### 2.3. Phase Solubility Studies of Cannabinoids According to the Types of Cyclodextrin

The phase solubility studies of the cannabinoids were performed per the method established by Higuchi and Connors [36]. Different α-CD, β-CD, γ-CD, HP-β-CD, and M-β-CD (0.625–10 mM) concentrations in distilled water were prepared. An excess amount of cannabis ethanol extraction 500 µL (264.85 mg/mL of THCA or 258.25 mg/mL CBDA) was added into a 5 mL CD solution. In addition, phase solubility studies of the cannabinoids with M-β-CD were performed in aqueous buffer. The mixture was mixed in a bath sonicator for 30 min and left for further mixing at 100 rpm and 25 ± 2 °C for 48 h in an incubator shaker. After equilibration, each sample suspension was centrifuged at 4500 rpm for 30 min. The ratios of ethanol and the pH of the suspensions were investigated to optimize the inclusion efficiency of the cannabinoids. Cannabinoid/CD inclusion complex was prepared using different ratios of ethanol (EtOH) to water (2.5, 5, 10, 15, and 20% EtOH(*v*/*v*)).

Supernatants with uncomplexed cannabinoids were filtered using a Polyvinylidene Fluoride (PVDF) 0.22 µm syringe filter. The filtrate was diluted and analyzed under validated HPLC conditions. The concentrations of solubilized cannabinoids were plotted against different concentrations of CD (mM). The slope and y-intercept (S_0_) of the straight lines for the phase solubility studies with different types of CDs were used to calculate the stability constant and complexation efficiency, as follows:

(1) $$\mathrm{Stability}\;\mathrm{constant};\;K_{s}=\frac{slope}{S_{0}(1-slope)}$$

(2) $$\mathrm{Complexation}\;\mathrm{Efficiency};\;\mathrm{CE}=\frac{slope}{\left(1-slope\right)}$$

### 2.4. Preparation of Cannabinoids/M-β-CD Inclusion Complex

In this study, the cannabinoids/M-β-CD complex was prepared using spray-drying, freeze-drying, and spray-freeze-drying methods. Each cannabinoid extract was dissolved in an aliquot of 15% ethanol and added to an aqueous solution of M-β-CD under shaking for 48 h at 120 rpm. Different molar ratios (1:1, 1:2, and 1:5) of cannabinoids and M-β-CD were weighed. M-β-CD was dissolved in 42.5 mL aqueous buffer pH 8.5–9.5. THCA or CBDA was dissolved in 7.5 mL ethanol, gradually added into the M-β-CD solution, and mixed using a magnetic stirrer for 48 h. After 48 h of incubation, the suspension was filtered by a 0.45 μm Nylon membrane filter. The filtrate was solidified by spray-drying, freeze-drying, and spray-freeze-drying. The inclusion complex was sieved and stored in a sealed container at 25 °C until further experiments.

Spray-drying was performed using a Büchi B-290 Mini Spray Dryer (Büchi Laboretechnik AG, Flawil, Switzerland). The cannabinoids/M-β-CD complex solution with a final solvent ratio of 85:15 (distilled water: ethanol) was used as the final composition. The processing conditions for spray-drying were set as follows: air inlet temperature at 150 °C, air outlet temperature at 80–95 °C, airflow rate of 600 L/h, speed of the peristaltic pump at 12.5 mL/min, and nozzle mesh size of 5.5 μm.

Freeze-drying was performed using a Dura Stop/Dura Dry MP Freeze Dryer (FTS Systems, New York, NY, USA). Sample formulations were frozen at −80 °C. The primary drying phase was programmed at a product temperature of −90 °C and a 48–55 mTorr pressure for 48 h.

The spray-freeze-drying technique used a single nozzle to release a fluid, immediately dried using liquid nitrogen. Each CD complex solution, through a nozzle with a mesh size of 5.5 μm, was directly atomized into liquid nitrogen. The fluidized bed system of the Niro Aeromatic Fielder MP-Micro (GEA, Duesseldorf, Germany) controlled the airflow. The pulsed frequency of spray was defined as the level of the pulse and spraying time in a spray cycle maintained at 5 min. The pulsed frequency ranged from 0 to 1, in which zero case indicated that the sample solution was sprayed continuously, and 1 means that the sample solution was sprayed for 1 min. Then, it was stopped for 1 min. The spray rate and atomization pressure ranges were 0.8–1.2 g/min and 10–20 psi, respectively. A peristaltic pump MP-2000A (EYELA, Bohemia, NY, USA) was used to control the flow rate of the suspended formulation. In this procedure, the droplets released from the nozzle above the boiling cryogenic liquid begin to freeze as they descend through a cold liquid nitrogen atmosphere. When these droplets were thermodynamically affected by cryogenic liquid, they were instantly frozen. After atomization, the remaining liquid nitrogen was evaporated, and the resulting powder was dried for 24 h in a Dura Stop/Dura Dry MP Freeze Dryer (FTS Systems, New York, NY, USA). The final step involved collecting the dried particles by sieving with 850 μm of No. 20 standard sieve. The freeze-dried or spray-freeze-dried inclusion complex powder was sieved and stored in a sealed container at 25 °C until further experiments.

### 2.5. Solid-State Characterization of Cannabinoids and Cyclodextrin Inclusion Complex

#### 2.5.1. Determination of Cannabinoid Solubility

Cannabinoid acids, including THCA or CBDA (equivalent to 1 mg) and cannabinoid acid/CD inclusion complex (equivalent to 1 mg cannabinoids), were dissolved in 5 mL of distilled water. The solutions were stirred on a shaker for 8 h and filtered through a 0.22 μm PVDF syringe filter (Milliporesigma, Oakville, ON, Canada). The sample filtrate was centrifuged (4500 rpm) for 20 min. Subsequently, the concentrations of cannabinoid acids in the different test samples were determined by HPLC, as described above.

#### 2.5.2. Field-Emission Scanning Electron Microscopy (FE-SEM)

The surface morphologies of the physical mixture, spray-dried, freeze-dried, and spray-freeze-dried CD inclusion complex were evaluated by field-emission scanning electron microscopy (FE-SEM) (JSM-6700F, JEOL, Tokyo, Japan). The sample powders were placed on carbon tape. The carbon tape was coated with gold for 2 min under a vacuum. The samples were visualized under an acceleration voltage of 5.0 kV.

#### 2.5.3. Differential Scanning Calorimetry (DSC)

The thermograms of the CD inclusion complex were obtained using a differential scanning calorimeter (DSC Q2000, TA instruments, New Castle, DE, USA). The samples were accurately weighed in a hermetic aluminum pan and compared to an empty pan. A sample containing approximately 5–7 mg of solid mass was weighed in an aluminum pan and sealed hermetically under an inert atmosphere (N_2_). The analysis was performed at heating and cooling rates of 10 K/min using an empty pan as a reference. The samples were heated from 10 to 250 °C, cooling to 25 °C.

#### 2.5.4. X-ray Diffraction (XRD)

The XRD patterns of the samples (cannabinoid ethanolic extracts, M-β-CD, cannabinoid acid/M-β-CD complex) were determined by a Philips X’Pert Pro diffractometer (Malvern Panalytical, Malvern, Worcestershire, UK) with Cu Kα radiation. The applied current and voltage were set to 100 mA and 40 kV, respectively. The scanning rate employed was 0.15°/min over a diffraction angle of 2θ in the range of 5–60°.

### 2.6. Proton Nuclear Magnetic Resonance (^1^H NMR) Spectroscopy

Proton nuclear magnetic resonance (^1^H NMR) spectroscopy was used to identify the inclusion process of cannabinoid acids on the M-β-CD. The ^1^H NMR spectra of the freeze- and spray-freeze-dried complexes were recorded using a Bruker Avance III 600 HD spectrometer (Bruker, Billerica, MA, USA). The samples (1 mg) were dissolved in D_2_O (1.0 mL) for the NMR analysis. Samples were prepared from cannabinoid acid/M-β-CD (1:2, molar ratio) in D_2_O and sonicated for 20 min.

### 2.7. Stability Studies of the Inclusion Complex in Simulated Physiological Conditions

The stability studies of cannabinoid acids (THCA, CBDA) and cannabinoid acid/M-β-CD spray-freeze-drying complex were performed in 10 mM PBS pH 7.4 at 37 °C ± 0.5 °C to simulate the physiological conditions. This solution was degassed in an ultrasonic bath for 1 h to eliminate oxygen, placed in glass vials, and incubated in a shaking incubator (agitation at 100 rpm) for 7 d. THCA, CBDA (in ethanolic extracts), and cannabinoids/M-β-CD complexes were diluted by PBS pH 7.4 to set as 1.0 μg/mL. At predetermined times (1, 2, 3, 4, 5, 6, and 7 d), aliquots were withdrawn and filtered by a 0.22 µm PVDF syringe filter. The samples were analyzed using the RP-HPLC method described in Section 2.2 to evaluate the assay of cannabinoid acids.

### 2.8. In Vitro Permeation Studies

In vitro permeation studies were performed using six Franz diffusion cells with an effective diffusion area of 1.767 cm^2^ (15.1 mm diameter orifice) to evaluate the release of cannabinoid acids from the CD inclusion complexes. Synthetic 0.22 μm PVDF membranes were hydrated in phosphate buffer (pH 7.4) at 25 °C for 1 h. The membrane was mounted between the donor and receptor cells. The receptor cell was filled with 12.0 mL of PBS buffer as a reception medium, and the medium was magnetically stirred at 600 rpm and maintained at 32 ± 0.5 °C with a circulating system. Cannabinoid/CD inclusion complex (0.5 g) or cannabinoid extracts were added to donor compartments. An amount of 0.5 mL of the receptor medium was withdrawn from the receptor cells at predetermined intervals of 0.25, 0.5, 1, 2, 4, 8, 16, and 24 h. Subsequently, the same volume of fresh receptor medium was added to maintain a constant volume of the receiving solution.

### 2.9. In Vitro Anti-Cancer Activity Assay Using Human Breast (MCF-7) Cancer Cell Lines

The cell lines were obtained from the University of Alberta Drug Development Innovation Center (DDIC). We have used the MCF-7 (ATCC HTB-22) breast cancer cell lines. These cell lines are ATCC cells (Manassas, VA, USA). The MCF-7 cell line was cultured in DMEM medium supplemented with 10% FBS, penicillin (100 U/mL), and streptomycin (100 μg/mL) and maintained at 37 °C with 5% CO_2_ in a humidified atmosphere. The cytotoxicity of cannabinoids/M-β-CD complexes was determined using the Alamar blue^®^ assay [37]. As the Alamar blue^®^ fluorescence intensity changes indicate the reduction action of resazurin, the active cell could mediate the metabolic activity of mitochondrial enzymes. Entering living cells, resazurin, the active ingredient of the Alamar blue^®^ reagent, was reduced to resorufin, a compound that changed it to red in color and to highly fluorescent. When cells were exposed to trypsin until completing the detachment at 37 °C for 20 min, cells were resuspended and counted (Neubauer chamber), diluted in culture media at a density of 2.5 × 10^4^ cells/mL, seeded onto 96-well microplates (100 μL per well; 2.5 × 10^3^ cells/well) and cultured for 24 h.

After that, culture media was removed and replaced by FBS-free culture media supplemented with the cannabinoids/M-β-CD formulation. The concentration of cannabinoid acid was set at 0.1, 1.0, 10.0, 100, 200, and 500 (µg/mL) and incubated for an additional 24 h. To determine the cell viability, 10% (*v*/*v*) Alamar blue^®^ was added to the medium, and the absorbance was monitored by Multiskan EX, Labsystems (Thermo Fisher Scientific, Waltham, MA, USA) at wavelengths of 570 nm and 620 nm after 4 h culture, per the manufacturer’s protocol, and at several timescale points as indicated in the results section. The IC_50_ values of CD complexes on the MCF-7 cancer cells were analyzed using GraphPad Prism software version 5.01 (San Diego, CA, USA) [38].

## 3. Results and Discussion

### 3.1. Phase Solubility Studies of Cannabinoids According to Types of Cyclodextrin

In a prior study cited as [35], the analytical validation of individual cannabinoid acids, like THCA and CBDA, was presented. The linearity, fitting equation, correlation coefficient, limit of detection (LOD), and limit of quantitation (LOQ) were specifically examined for these cannabinoid acids. Additionally, this study assessed the precision and accuracy of quantifying THCA and CBDA. The correlation coefficient (R^2^) of THCA and CBDA determination were, respectively, 0.9998 and 0.9999. The calibration curves of cannabinoid acid were set using 10 concentrations of the response ratio over the range of 0.1953 µg/mL to 100.0000 µg/mL. The limit of detection of THCA and CBDA was 0.0650 µg/mL. This limit was validated by injecting samples with known low concentrations near the LOQ in triplicates. The precision and accuracy were within acceptable limits (RSD ≤ 2%).

**Figure 1:**
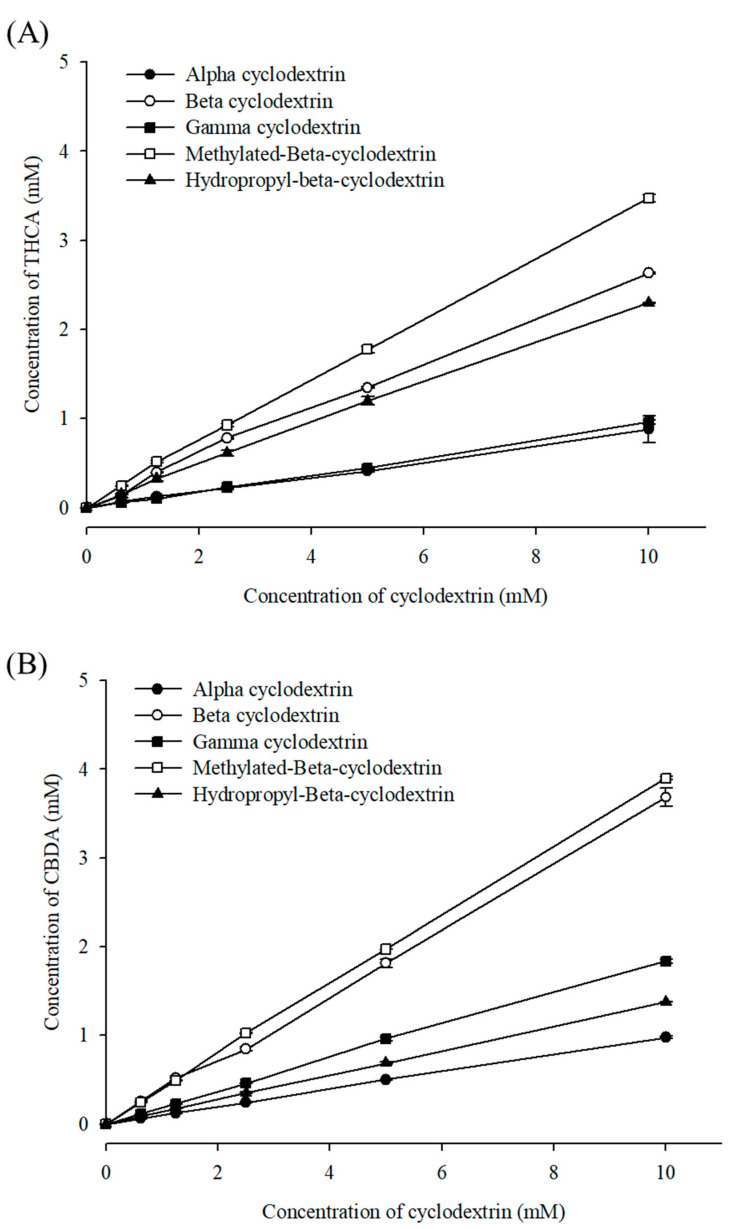
Phase solubility plots of (**A**) THCA and (**B**) CBDA in different types of cyclodextrins, mean ± SD (n = 3).

Figure 1 shows a phase solubility plot of the cannabinoid acids in the five CDs. The plot depicts the Higuchi A_L_-type phase solubility for all complexes and a linear increase in cannabinoid solubility with increasing CD concentration. Table 1 summarizes the complexation parameters for all cannabinoid acid/CD inclusion complexes. As previously reported, the stability constant (K_S_) was used to determine the inclusion efficiency of CDs in drugs [39,40,41].

The solubilizing efficiency of cannabinoids in different CDs are in the following order: M-β-CD > β-CD > HP-β-CD > γ-CD > α-CD. The K_s_ values corresponded to greater solubilization of THCA at 10 mM M-β-CD (3.47 ± 0.04 mM) compared to the systems containing β-CD and HP-β-CD, with values of 2.63 ± 0.01 and 2.31 ± 0.05 mM, respectively. In the case of CBDA, the complexation efficiency of M-β-CD was also higher than β-CD. Additionally, all five inclusion complexes showed improved solubility of cannabinoids in aqueous solution compared to cannabinoids alone. Considering the preliminary study for a 2% (*w*/*v*) CD complex of cannabinoids for 48 h, M-β-CD was highly effective for encapsulating THCA.

**Figure 2:**
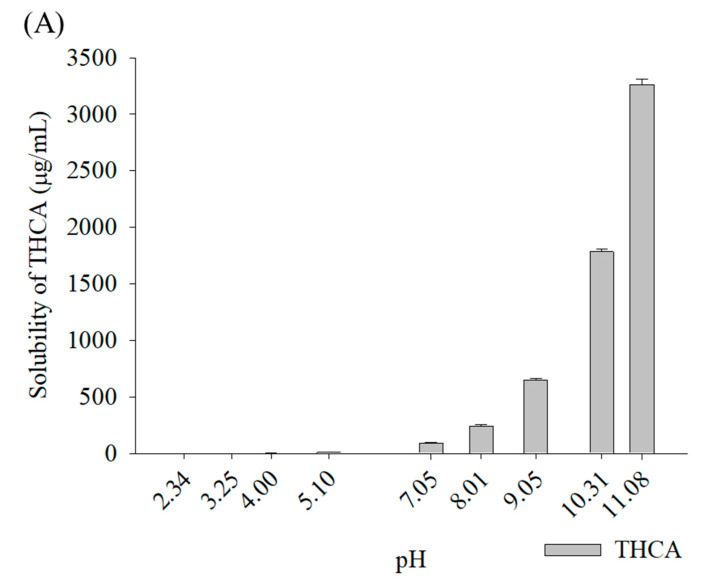

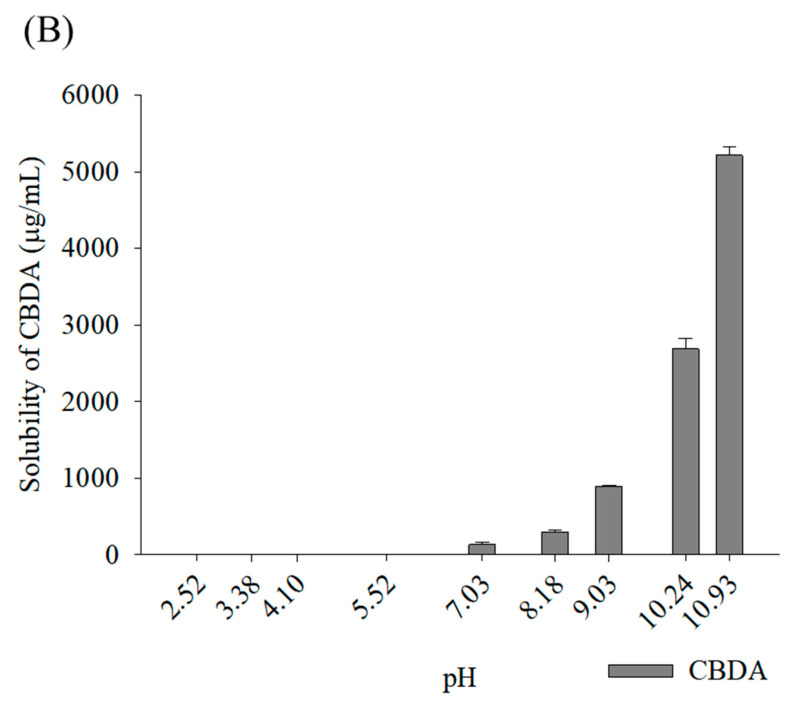
pH-dependent phase solubility of (**A**) THCA and (**B**) CBDA with M-β-CD in aqueous buffer solution, mean ± SD (n = 3).

As shown in Figure 2, the solubility of THCA with M-β-CD at pH 7.45 was increased 11.1-fold, like that at pH 5.10. Additionally, the pH-dependent solubility of CBDA with M-β-CD showed a significant improvement of 13.3-fold at pH 7.03 compared to that at pH 5.52. Therefore, the acid-base titration results indicated that the complexation efficiency of the THCA/M-β-CD inclusion complex could be improved by adjusting the pH of the solution.

### 3.2. Solid-State Characterization of Cannabinoids and CD Inclusion Complex

#### 3.2.1. Solubility Determination

According to the ratio of EtOH/water for the CD complexation efficiency of cannabinoid acid, the loading contents and feasibility of cannabinoid acids/M-β-CD inclusion complex summarized in Table 2, the percentage of ethanol was set to 15% (*v*/*v*) for the preparation method.

Several methods can be used to prepare the CD–guest inclusion complex. The cannabinoids/M-β-CD complex was prepared by spray-drying, freeze-drying, and spray-freeze-drying methods. The molar ratio of cannabinoid/M-β-CD was optimized by comparing the aqueous solubility of each cannabinoid acid in different dried CD forms. The preparation methods varied the solubility of THCA and CBDA in the M-β-CD inclusion complex. As summarized in Table 3, the molecular ratio of cannabinoids/M-β-CD varied the solubility of THCA and CBDA in the M-β-CD inclusion complex.

Interestingly, the solubility determination of cannabinoid acid indicated that the processing condition affected the complexation efficiency of each cannabinoid (spray-dried form). When an initial molar ratio of 1:1 was set, the aqueous solubility of THCA and CBDA spray-drying CD complex was 220 ± 9 μg/mL and 300 ± 70 μg/mL, respectively. As the molar ratio of cannabinoid acid/M-β-CD increased to 1 to 2, the water solubility of cannabinoid acid in the M-β-CD inclusion complex (freeze-dried form) was also increased from 78 ± 5 μg/mL to 190 ± 10 μg/mL. However, the 1 to 5 molar ratio did not improve the solubility of each cannabinoid acid in freeze-dried form. When an initial molar ratio of 1:2 (cannabinoid acid: M-β-CD) was selected, the water solubilities of THCA and CBDA in spray-freeze-drying CD complex were 190 ± 10 μg/mL and 200 ± 20 μg/mL, respectively. Regardless of preparation methods for CD inclusion complexes, the solubilities of the cannabinoid acids decreased when the M-β-CD had a relatively higher molar ratio of 1 cannabinoid acid to 5 CD molecules. Among the weight ratios of the drug-to-CD complex combinations, a 1:2 ratio was the most efficient in forming the complex, suggesting its potential to exhibit the highest solubility.

Considering the drying process of CD complexes, the spray-freeze-drying method could stably load the cannabinoid acid with a high solubilizing effect. The solubility of THCA increased to 930 ± 20 μg/mL in the spray-freeze-dried cannabinoid acid/M-β-CD complex. As the molar ratio was 1:2, the solubility of THCA in the spray-freeze-dried inclusion complex increased from those of spray-dried CD form (480 ± 20 μg/mL) and freeze-dried CD form (190 ± 10 μg/mL) to 930 ± 20 μg/mL. At the same molar ratio (1:2 = cannabinoid acid: M-β-CD molecule), the THCA solubility in the spray-freeze-dried CD complex showed 4.2-fold higher than that of the freeze-dried CD complex. As a result, the spray-freeze-drying method was the most effective technique for stabilizing cannabinoid acids and improving their aqueous solubility in the CD inclusion complex.

#### 3.2.2. FE-SEM Analysis

**Figure 3:**
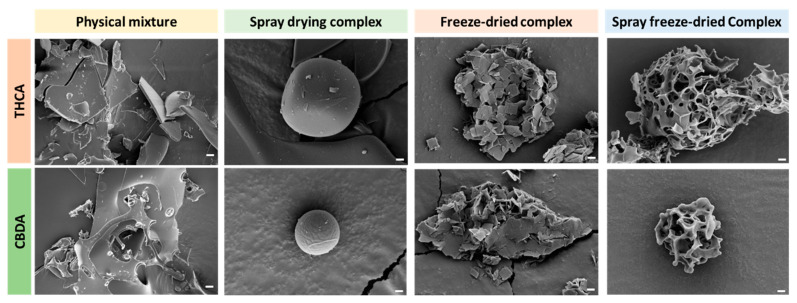
FE-SEM images of cannabinoids/M-β-CD inclusion complex using different preparation methods (Scale bar: 2 µm).

Field-emission scanning electron microscopy (FE-SEM) was used to visualize the surfaces of the solid powders for morphological examination. As shown in Figure 3, the preparation method affected the morphology, with remarkable CD molecules on the surface of the powder. The shapes of the physical mixtures showed that they mainly comprised CD molecules, and the cannabinoids were partially mixed and adsorbed onto the CDs.

#### 3.2.3. DSC Analysis

**Figure 4:**
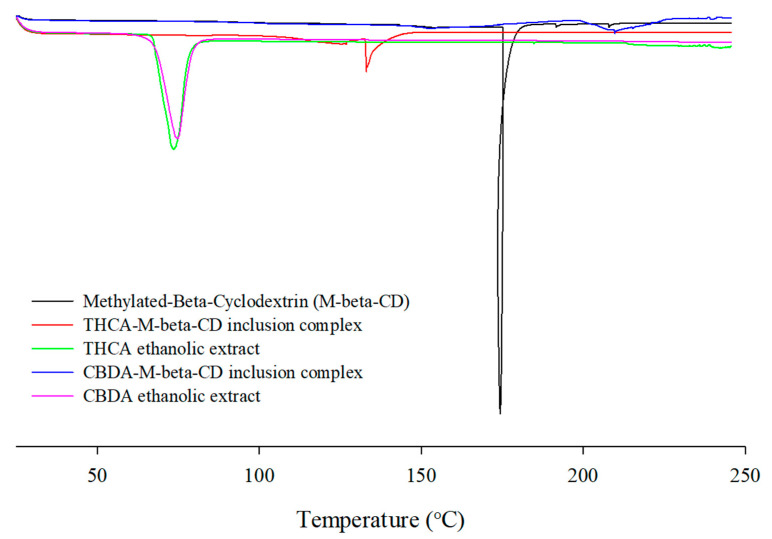
DSC thermograms of M-β-CD, cannabinoids ethanolic extracts, and cannabinoids/CD inclusion complex.

DSC studies have characterized the thermograms of CD and cannabinoid acids, with a successful inclusion complex indicated by either the absence of a peak or any shift from the peak patterns at different temperatures [42,43]. As shown in Figure 4, the thermogram of M-β-CD showed a sharp endothermic peak at 181.2 °C and an exothermic peak at 225.0 °C, suggesting degradation. However, the physical mixture slightly shifted the peaks, suggesting a weak interaction between the cannabinoids and M-β-CD. Thermograms of the M-β-CD complex showed two endothermic peaks, whereas the characteristic peak of cannabinoid acid was absent, indicating the successful inclusion of THCA or CBDA in the cavity of M-β-CD. The shift in the endothermic peaks observed in the THCA/M-β-CD complex indicated possible interactions between the cannabinoids and CD molecules owing to the inclusion process during complex formation.

#### 3.2.4. XRD Analysis

**Figure 5:**
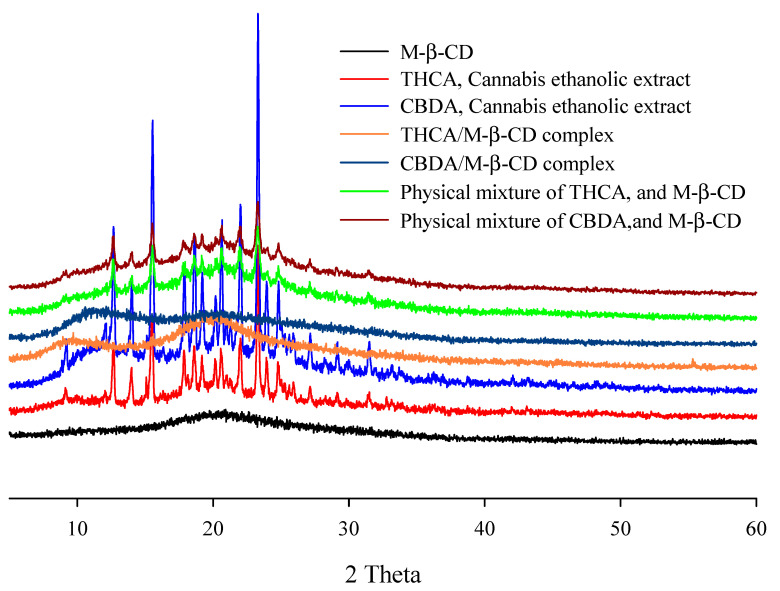
XRD patterns of M-β-CD, cannabinoids ethanolic extracts, cannabinoids/cyclodextrin inclusion complex, and physical mixtures.

The solid-state of the cannabinoid acid in the CD complex was investigated by the XRD patterns of each sample. As shown in Figure 5, the THCA and CBDA ethanolic extract showed a crystalline structure, as demonstrated by a sequence of sharp diffraction peaks between 5 and 60°. In the case of the cannabis ethanolic extract, the characteristic peaks of each cannabinoid acid were maintained. Additionally, the physical mixtures maintained the specific patterns of the cannabinoid acid (diffraction peaks at 15.5°, 17.5°, and 23.3°) and M-β-CD (halo pattern). If the guest molecule was not successfully included in the CD complex, the crystallinity pattern of each ingredient would be exhibited in the XRD diffractogram. For example, the reported XRD study for determining CD complexation using emodin as a guest molecule disproved that the crystallinity of each ingredient has been converted to an amorphous form [39]. These results indicated that the inclusion complex of cannabinoid acid could form the non-crystalline state of drug formulation after incorporation into the CD molecule. The amorphization and absence of crystallinity of the active ingredients caused by the CD complexation align with the results presented in other studies.

### 3.3. ^1^H NMR Studies of Cannabinoids/M-β-CD Inclusion Complex

**Figure 6:**
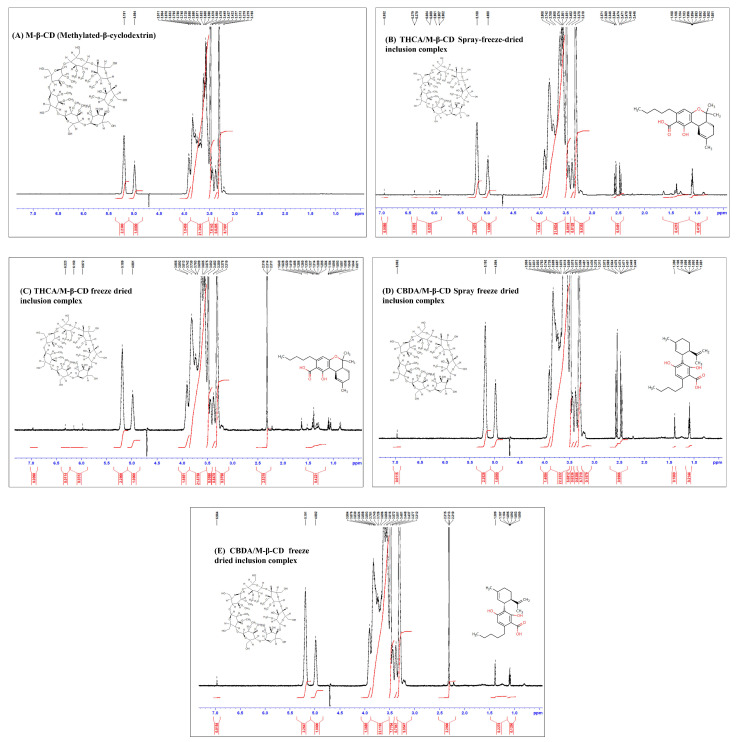
^1^H NMR spectra of M-β-CD and cannabinoid acid/CD inclusion complex prepared by the freeze-dried or spray-freeze-dried method (see also enlarged version of spectra in Figure S1). (**A**) M-β-CD, (**B**) THCA/M-β-CD spray-freeze-dried inclusion complex, (**C**) THCA/M-β-CD freeze-dried inclusion complex, (**D**) CBDA/M-β-CD spray-freeze-dried inclusion complex, and (**E**) CBDA/M-β-CD freeze-dried inclusion complex.

The inclusion complexation between cannabinoid acid and M-β-CD in solution was investigated by ^1^H NMR spectroscopy. The inclusion complexation of cannabinoid acid and CDs was further confirmed by examining the changes in the chemical shifts in ^1^H NMR spectra. Figure 6 shows the ^1^H NMR spectra of M-β-CD and cannabinoid acid/CD inclusion complex prepared by the freeze-dried or spray-freeze-dried method. The enlarged version of ^1^H NMR spectra is also available in Figure S1. The ^1^H NMR spectra of cannabinoid acids showed structural similarities and significant signal overlaps. Particularly, strongly shielded proton signals such as alkyl protons were mainly detected in the high field of the spectrum (approximately 0–3 ppm). In this region, the proton signals from the C5 or C3 alkyl side chain of the cannabinoids were exhibited. The protons of THCA and CBDA displayed chemical shift values in the range of 5.7 to 7.0 ppm and 0.5 to 2.7 ppm, respectively. The ^1^H NMR spectra of M-β-CD were consistently observed in all inclusion complex formulations. Additionally, the ^1^H NMR spectra of freeze-dried cannabinoid acid/CD complex exhibited two distinct peaks in the 2.4–2.7 ppm range, corresponding to the aromatic methyl group. Inclusion compound formation is a new substance in which high shift changes of H-3 and H-5 protons reflected the location of cannabinoid acid into the cavity of M-β-CD. Therefore, these spectra suggested that the inclusion of guest molecule cannabinoids to host molecule M-β-CD depended on the molecular arrangement displaced in a specific hydrophobic moiety.

### 3.4. In Vitro Permeation Profiles of Cannabinoids/M-β-CD Inclusion Complex

**Figure 7:**
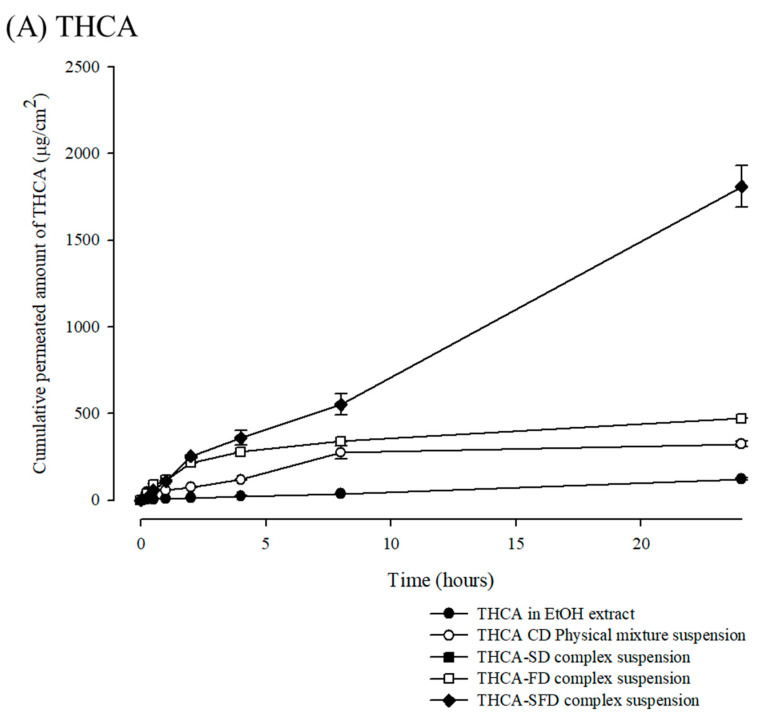

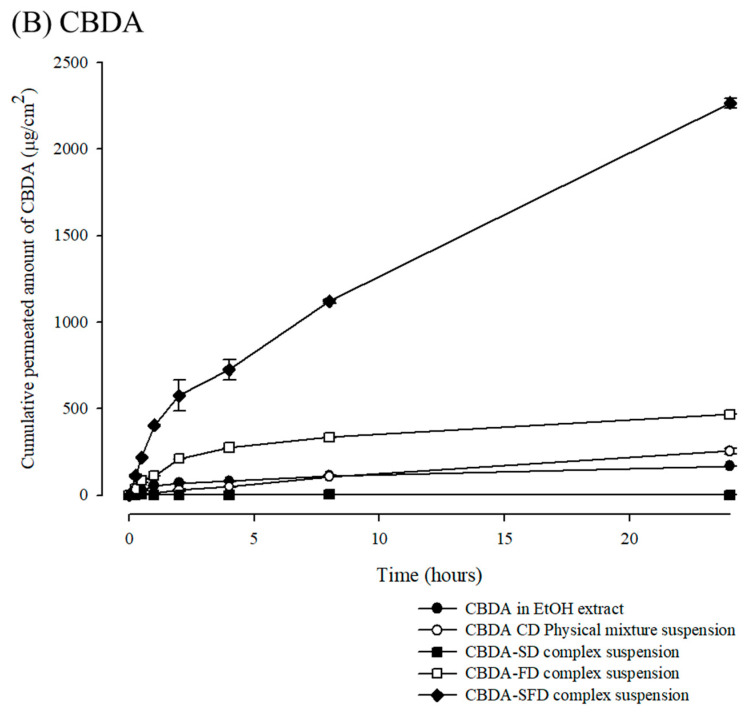
In vitro permeation profiles of THCA (**A**) or CBDA (**B**) from cannabinoid acids/CD inclusion complex suspension system.

The permeability profiles of the cannabinoid acids were evaluated using in vitro Franz cells with PBS (pH 7.4) as the receiver medium. Figure 7 shows the in vitro permeation profiles of THCA or CBDA from cannabinoid acids/CD inclusion complex suspension system across the membrane over time. The permeation profiles of the THCA- and CBDA-CD inclusion complex exhibited sigmoidal patterns with two phases. The initial phase demonstrates a gradual increase in the permeation profile of the cannabinoid acids. In the later phase, the permeation profiles fit a zero-order model, as indicated by the high degree of correlation.

Notably, there were significant differences between the permeation profiles and Q_6h_ values of the cannabinoid-CD-complex suspension. Cannabinoid acids were highly lipophilic molecules with a calculated log Kow of approximately 8, and their permeation was assumed to be passive diffusion through a transcellular pathway. The resulting spray-freeze-dried powders were suspended in a dispersion that might have a lower partition into the synthetic membrane. The constant flux of permeation of cannabinoid acid from the spray-freeze-drying complex was attributed to the complexation of the cannabinoid acid covering the molecule inside. CD molecules were spontaneously positioned when thermodynamically placed in a pH-adjusted aqueous medium [44].

Several studies have proven that the permeation efficiency of cannabinoids was determined by correlating the lipophilicity of each cannabinoid and the permeation pathway of the compound [45]. In recent investigations, a small number of cannabinoids could permeate through the mucosa or skin, whereas a finite amount was deposited inside the permeated membrane [46,47]. It was reported that the overuse of solubilizing agents could occasionally induce mucosal irritation and permeation obstruction [48,49]. These challenges could negatively affect the permeation of lipophilic cannabinoids. CD derivatives could stably permeate lipophilic compounds that pass through the biological membrane via the paracellular route [50,51,52]. CD molecules could act as organic cosolvents to induce the molecular mobility of lipophilic molecules, maximizing the thermodynamic activity [53]. As a result, the complexation of cannabinoid acid in CDs could be an alternative way to reduce the adverse effects of surfactant overuse.

### 3.5. Stability Profiles of Cannabinoid Acids and CD Inclusion Complex in Simulated Physiological Condition

**Figure 8:**
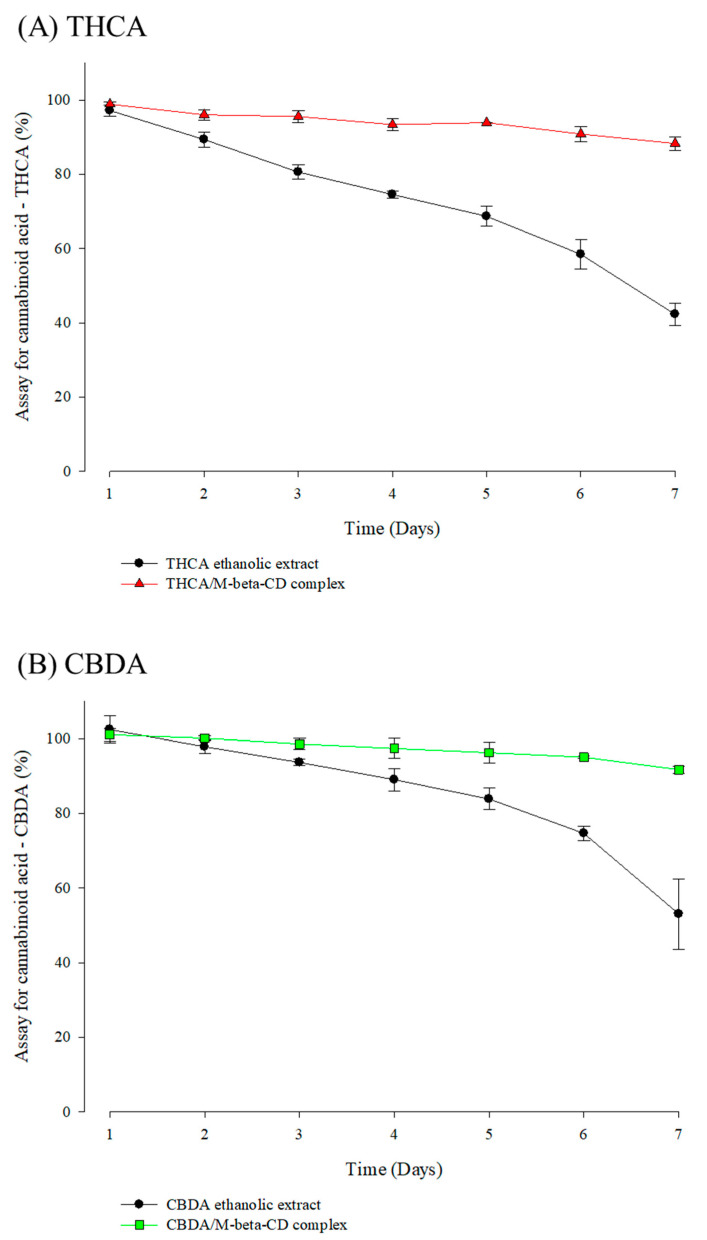
Stability profiles of cannabinoid acids in simulated physiological conditions (**A**) THCA and (**B**) CBDA.

THCA and CBDA in cannabis ethanolic extracts are highly decarboxylated into other THC and CBD [54]. In the case of the simulated physiological conditions (pH 7.4 and 37 ± 0.5 °C), 10% (*w*/*w*) of the cannabinoid acid was degraded within the first day of this study. The stability profiles of cannabinoid acid and its M-β-CD complex in a buffer solution (pH 7.4) are shown in Figure 8. Both cannabinoid acids (THCA and CBDA) maintained their drug contents in the M-β-CD inclusion complex within 7 d. In fact, cannabinoid acid contents in cannabis ethanolic extracts decreased by approximately 50% in this condition, whereas the contents of each cannabinoid acid were above 90% in cannabinoid acid/M-β-CD complex for 7 d. These results suggested that CD complexation could effectively entrap cannabinoids under simulated physiological conditions. Therefore, M-β-CD complexation could provide resistance for the model cannabinoid acid to physiological conditions compared to the cannabinoid acid alone.

### 3.6. In Vitro Anti-Cancer Activities of Cannabinoids/M-β-CD Inclusion Complex

**Figure 9:**
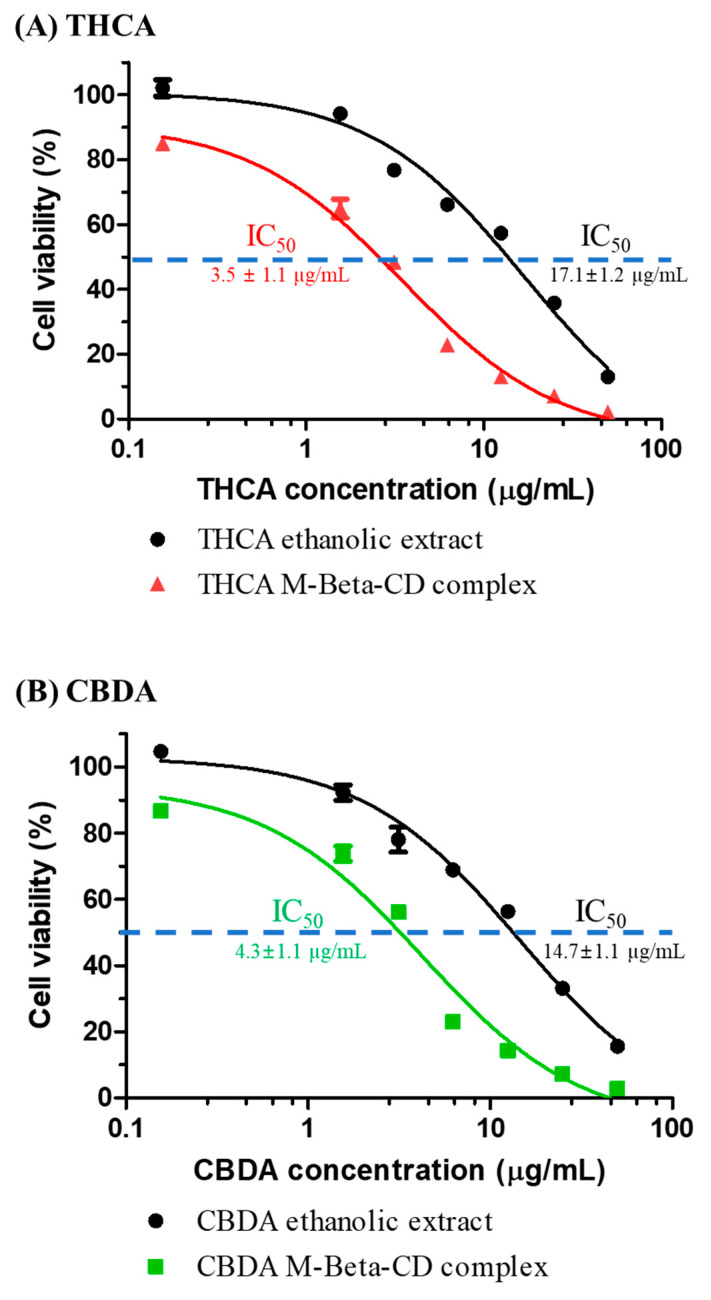
In vitro cytotoxicity of cannabis ethanolic extracts and cannabinoids/cyclodextrin inclusion complex (**A**) THCA and (**B**) CBDA.

The cytotoxicity of cannabinoid acids and cannabinoid acid/M-β-CD complex on the human breast (MCF-7) cancer cell line was evaluated using the Alamar blue^®^ assay. As shown in Figure 9, both the ethanolic extracts of cannabinoids and their inclusion complexes reduced the viability of MCF-7 breast cancer cells in a dose-dependent manner. A significant decrease in the viability of MCF-7 cancer cells was observed at 6.25 µg/mL of cannabinoid acid compared to lesser concentrations. When the concentration was 1.56 µg/mL, THCA or CBDA exhibited minimal cytotoxicity (below 15%) to these cancer cells.

Interestingly, the cannabinoid acid/M-β-CD complex slightly enhanced the anti-cancer effects on cell viability compared to cannabinoid acid alone. The IC_50_ (mean concentration that indicated 50% cell inhibition) of cannabinoid acid was 17.1 ± 1.2 μg/mL (THCA) and 14.7 ± 1.1 μg/mL (CBDA), whereas the IC_50_ of cannabinoid acid/M-β-CD complex decreased to 3.5 ± 1.1 μg/mL (THCA) and 4.3 ± 1.1 μg/mL (CBDA), respectively. There was approximately a 4-fold decrease in IC_50_ concentration upon the complexation of cannabinoid acid. Therefore, the anti-cancer effects of cannabinoid acid/M-β-CD inclusion complex was 3-fold more potent than the cannabinoid acid itself.

## 4. Conclusions

This study successfully demonstrated the potential of CD complexation to enhance the solubility and stability of cannabinoid acids (THCA and CBDA). Phase solubility studies determined the optimal stoichiometry of the inclusion complex. We specifically chose M-β-CD for our study due to its superior complexation efficiency with cannabinoid acid, potentially reducing the required dose of excipient in the final formulation. Among the various drying methods, the spray-freeze-drying method was the most effective for obtaining the desired cannabinoid acid/M-β-CD inclusion complex. These approaches addressed the optimum ratio and manufacturing process regarding the CD complexation of THCA and CBDA. Solid-state characterization techniques such as DSC, XRD, and FE-SEM analysis confirmed the non-crystalline state of the cannabinoid acid/CD inclusion complex. Chemical shift changes in ^1^H NMR spectroscopy supported evidence of the inclusion complex between cannabinoid acid and M-β-CD as a molecularly new substance in solution. Notably, the spray-freeze-dried powder of cannabinoid acids/M-β-CD inclusion complex exhibited superior permeation and stability profiles under simulated physiological conditions compared to the cannabis ethanolic extracts. In vitro cytotoxicity studies demonstrated that spray-freeze-dried cannabinoids/M-β-CD complex had better anti-cancer activity than cannabinoids alone. Considering these findings, the CD complexation could be a promising option for developing cannabis-derived drug products.

## Figures and Tables

**Table 1:** Complexation parameters of cannabinoid acid in different types of cyclodextrin.

Types of Cannabinoid Acid	Types of Cyclodextrin	Slope	Y-Intercept (S_o,_ mM)	Stability Constant (K_S_, M^−1^)	Regression Coefficient (R^2^)	Complexation Efficiency (%)	Molar Ratio (Cannabinoids:CD)
THCA	α-CD	0.086	0.052	642	0.998	9.43	1.00:11.39
	β-CD	0.262	0.359	2140	0.997	35.5	1.00:3.80
	γ-CD	0.096	0.109	1280	0.998	10.6	1.00:10.38
	M-β-CD	0.344	0.448	2360	0.999	52.5	1.00:2.88
	HP-β-CD	0.227	0.241	1400	0.999	29.4	1.00:4.35
CBDA	α-CD	0.098	0.018	191	0.998	10.8	1.00:10.27
	β-CD	0.115	0.018	159	0.997	13.0	1.00:8.64
	γ-CD	0.185	0.035	199	0.998	22.6	1.00:5.45
	M-β-CD	0.390	0.141	420	0.999	63.9	1.00:2.55
	HP-β-CD	0.138	0.023	172	1.000	15.9	1.00:7.25

**Table 2:** The drug loading contents and molar ratio of THCA and CBDA in cannabinoid acids/M-β-CD inclusion complex according to the ratio of EtOH/water.

Types of Cannabinoids	Ratio of EtOH/Water	Inclusion Complex	
		Drug Loading Content (%)	Molar Ratio
THCA	20% EtOH (*v*/*v*)	11.05 ± 0.02	1:1.93
	15% EtOH (*v*/*v*)	11.18 ± 0.03	1:1.91
	10% EtOH (*v*/*v*)	8.37 ± 0.01	1:2.62
	5% EtOH (*v*/*v*)	4.98 ± 0.02	1:4.58
	2.5% EtOH (*v*/*v*)	2.38 ±0.04	1:9.82
CBDA	20% EtOH (*v*/*v*)	10.51 ± 0.05	1:2.27
	15% EtOH (*v*/*v*)	12.25 ± 0.09	1:1.72
	10% EtOH (*v*/*v*)	5.34 ± 0.01	1:4.26
	5% EtOH (*v*/*v*)	2.83 ± 0.00	1:8.25
	2.5% EtOH (*v*/*v*)	1.02 ± 0.01	1:23.28

Data were given as mean ± SD, n = 3.

**Table 3:** The aqueous solubility of cannabinoids in M-β-CD inclusion complex per the preparation method.

**Preparation Method**	**Types of Cannabinoids**	**Aqueous Solubility of Cannabinoid Acids (µg/mL)**		
		**Molar Ratio (Cannabinoids: M-β-CD)**		
		**1:1**	**1:2**	**1:5**
Spray-drying	THCA	220 ± 9	480 ± 20	338 ± 3
	CBDA	300 ± 70	554 ± 4	440 ± 10
Freeze-drying	THCA	78 ± 5	190 ± 10	110 ± 30
	CBDA	93 ± 8	200 ± 20	90 ± 10
Spray-freeze-drying	THCA	310 ± 20	930 ± 20	470 ± 60
	CBDA	450 ± 40	1090 ± 40	540 ± 60

Data given as mean ± SD, n = 3.

## Data Availability

The data can be shared up on request.

## Supplementary Materials

The following supporting information can be downloaded at https://www.mdpi.com/article/10.3390/pharmaceutics15112533/s1, Table S1. pH-dependent solubility of (A) THCA and (B) CBDA with M-β-cyclodextrin complex; Figure S1: Enlarged version of ^1^H NMR spectra with (A) M-β-CD, (B) THCA/M-β-CD Spray-freeze-dried inclusion complex, (C) THCA/M-β-CD freeze dried inclusion complex, (D) CBDA/M-β-CD Spray-freeze-dried inclusion complex, and (E) CBDA/M-β-CD freeze dried inclusion complex.

## Abbreviations

CD	cyclodextrin
α-CD	α-cyclodextrin
β-CD	β-cyclodextrin
γ-CD	γ-cyclodextrin
HP-α-CD	hydroxypropyl-α-cyclodextrin
M-β-CD	methylated-β-cyclodextrin;
DSC	differential scanning calorimetry
FE-SEM	field-emission scanning electron microscopy
1H NMR	proton nuclear magnetic resonance spectroscopy
THC	tetrahydrocannabinol
CBD	cannabidiol
THCA	Tetrahydro-cannabinolic acid
CBDA	cannabidiolic acid
COX	cyclooxygenase
RP	reversed-phase
HPLC	high-performance liquid chromatographic system
PVDF	Polyvinylidene Fluoride
