# Supplementary material for: Investigation of Cannabinoid Acid/Cyclodextrin Inclusion Complex for Improving Physicochemical and Biological Performance
Source: Pharmaceutics. 2023 Oct 26;15(11):2533. doi: 10.3390/pharmaceutics15112533 (PMC10675134; doi:10.3390/pharmaceutics15112533)
Supplement: Supplementary file 1 [file pharmaceutics-15-02533-s001.zip › pharmaceutics-2672199-supplementary.pdf]

Table S1. pH-dependent solubility of (A) THCA and (B) CBDA with M-β-CD complex

(A) THCA

pH	The solubility of THCA (μg/mL)
2.34	Not detected
3.25	Not detected
4.05	1.2 ± 0.3
5.10	8.0 ± 0.2
7.05	91 ± 4
8.01	240 ± 10
9.05	650 ± 10
10.31	1780 ± 20
11.08	3260 ± 50

(B) CBDA

pH	The solubility of CBDA (μg/mL)
2.52	Not detected
3.38	Not detected
4.10	2.3 ± 0.2
5.52	10.3 ± 0.4
7.03	140 ± 20
8.18	300 ± 20
9.03	890 ± 10
10.24	2690 ± 130
10.93	5220 ± 110

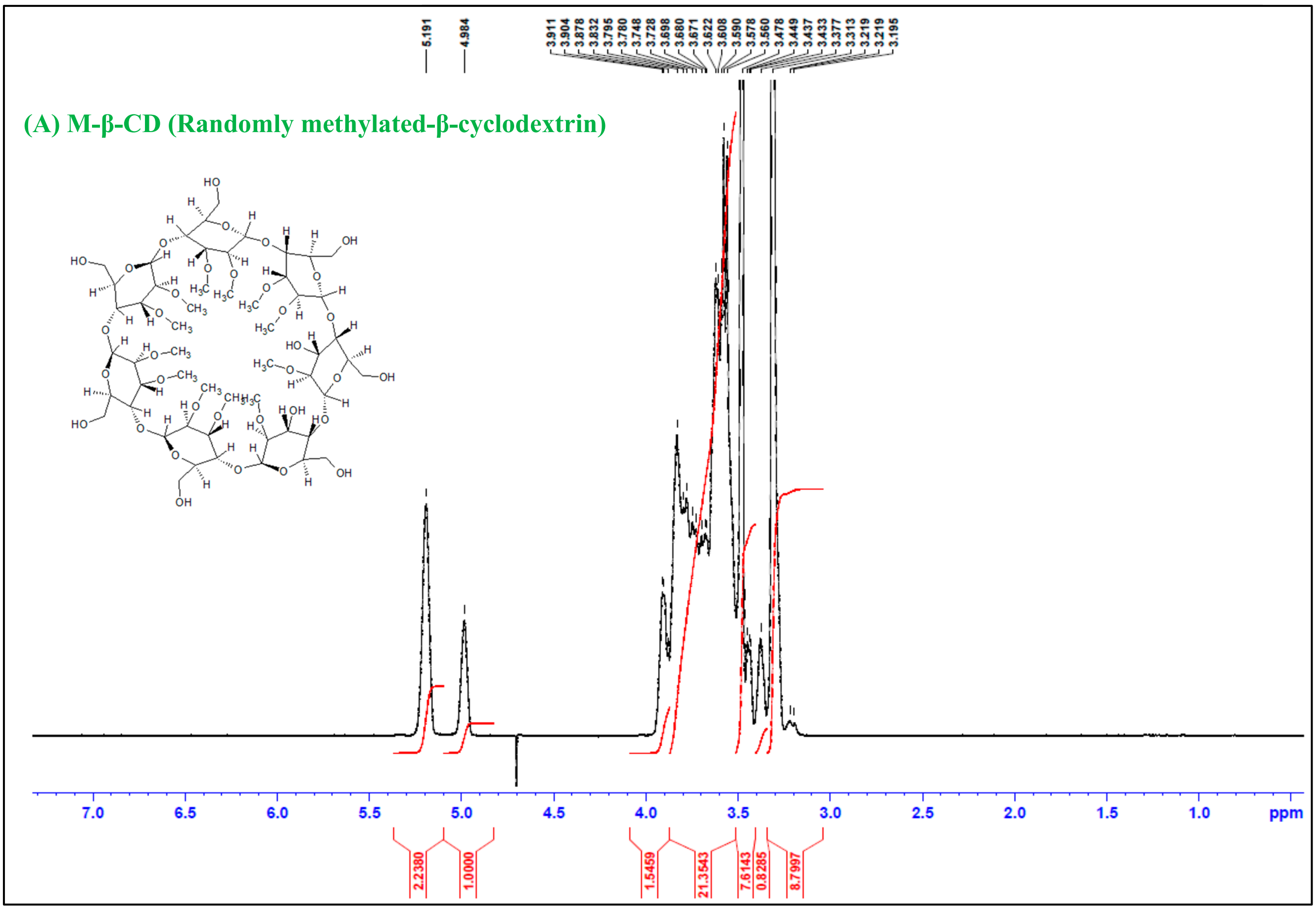
(A) M-β-CD (Randomly methylated-β-cyclodextrin)
ppm

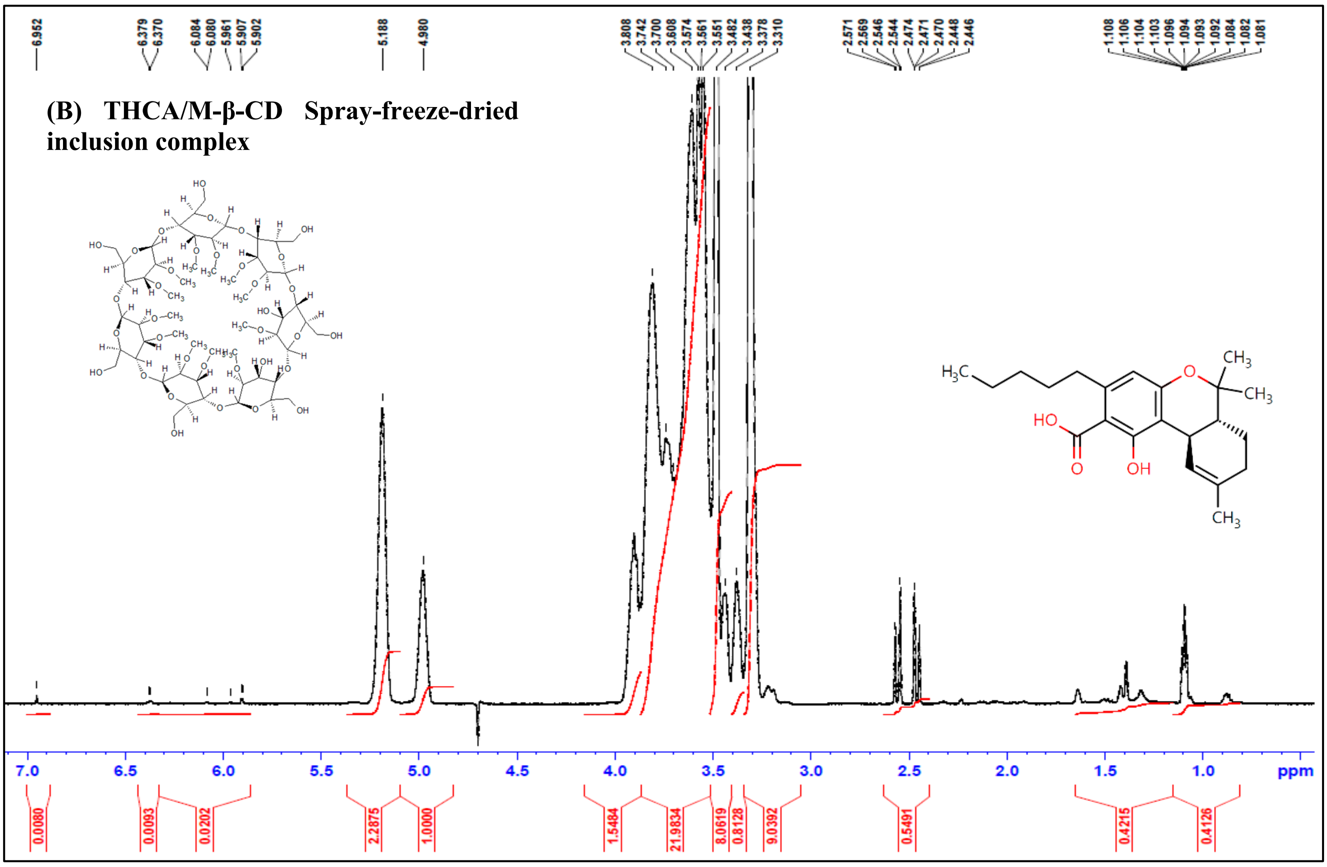
(B) THCA/M-β-CD Spray-freeze-dried inclusion complex
ppm

(C) THCA/M-β-CD freeze dried inclusion complex

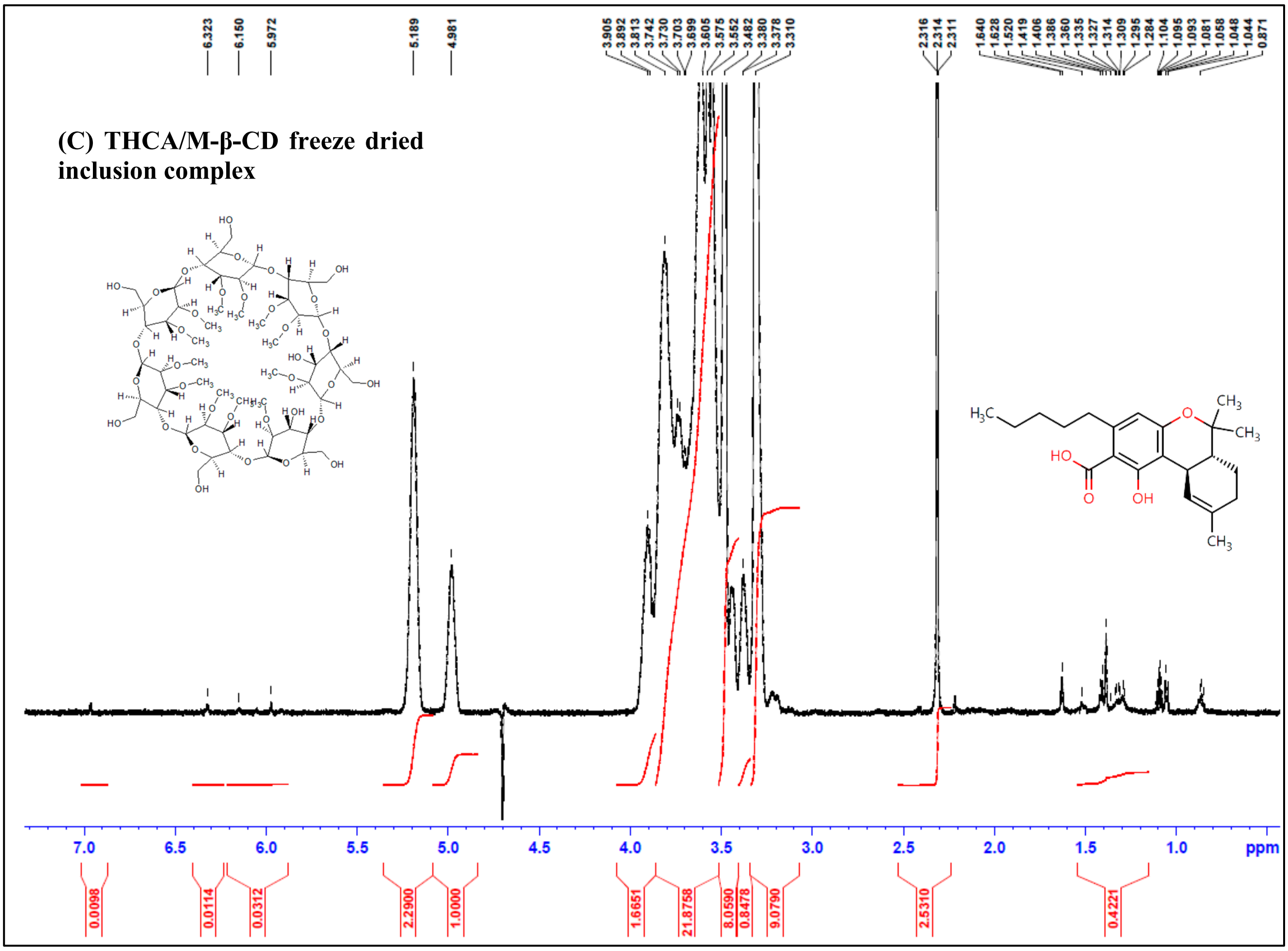

(D) CBDA/M-β-CD Spray freeze dried inclusion complex

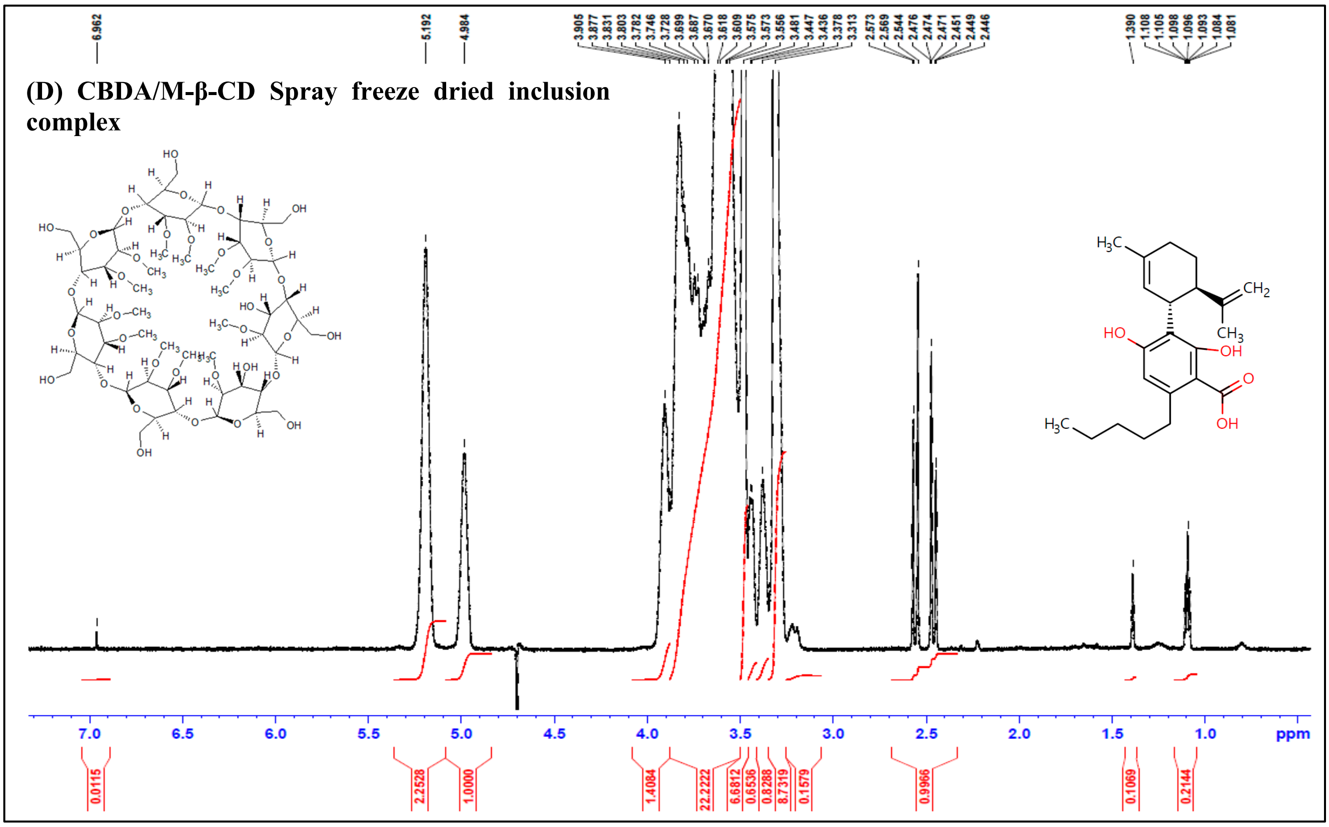

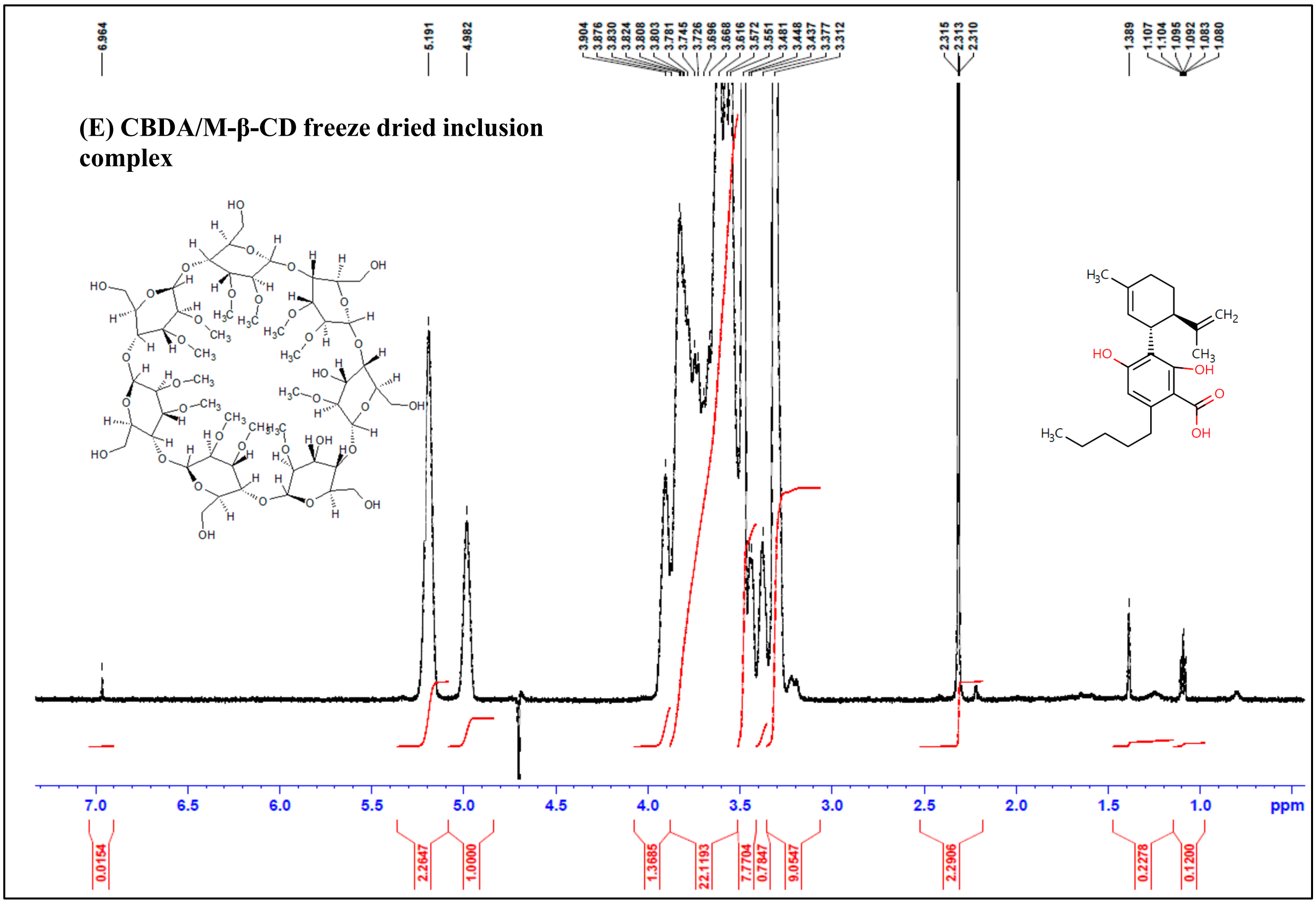

Figure S1. Enlarged version of ^{1}H NMR spectra with (A) M-**β**-CD, (B) THCA/M-**β**-CD Spray freeze dried inclusion complex, (C) THCA/M-**β**-CD freeze dried inclusion complex, (D) CBDA/M-**β**-CD Spray freeze dried inclusion complex, and (E) CBDA/M-**β**-CD freeze dried inclusion complex
